# Acid-sensing ion channel-1 contributes to the failure of myelin sheath regeneration following spinal cord injury by transcellular delivery of PGE2

**DOI:** 10.1186/s11658-024-00672-9

**Published:** 2024-12-03

**Authors:** Zuomeng Wu, Tianyu Han, Yixiang Dong, Wang Ying, Huang Fang, Yunlei Liu, Peiwen Song, Cailiang Shen

**Affiliations:** 1 Department of Orthopedics (Spinal Surgery), The First Affiliated Hospital of Anhui Medical University, Hefei, 230032 People’s Republic of China; 2 Laboratory of Spinal and Spinal Cord Injury Regeneration and Repair, The First Affiliated Hospital of Anhui Medical University, Hefei, 230032 People’s Republic of China; 3 Department of Medical Imaging, The First Affiliated Hospital of Anhui Medical University, Hefei, China; 4 Department of Orthopedics (Spinal Surgery), The First Affiliated Hospital of USTC, Hefei, 230032 People’s Republic of China; 5 Department of Clinical Laboratory, The First Affiliated Hospital of Anhui Medical University, Hefei, 230032 People’s Republic of China

**Keywords:** Spinal cord injury, Neural stem cells, Acid-sensing ion channel, Prostaglandin endoperoxide synthase 2

## Abstract

**Background:**

Traumatic injuries to spinal cord lead to severe motor, sensory, and autonomic dysfunction. The accumulation of inhibitory compounds plays a pivotal role in the secondary damage to sparing neural tissue and the failure of axonal regeneration and remyelination. Acid-sensing ion channel-1(ASIC1A) is widely activated following neurotrauma, including spinal cord injury (SCI). However, its role in SCI remains elusive.

**Methods:**

The effects of acidic environment on the differentiation and genes changes of neural stem cells (NSCs) were assessed by immunofluorescence staining and RNA-sequencing analysis, respectively. The expression of ASIC1A and prostaglandin endoperoxide synthase 2 (PTGS2) were detected by western blot and immunofluorescence staining. The concentration of prostaglandin E2 (PGE2) within NSC-derived extracellular vesicles were evaluated by ELISA. Small-interfering RNAs (siRNAs) were used to knock down Asic1a and Ptgs2 expression in NSCs. The myelin sheath regeneration and axonal remyelination in rats and Asic1a-KO mice were assessed by immunofluorescence staining.

**Results:**

Following injury to the spinal cord, ASIC1A was found to be colocalized and upregulated in NSCs. ASIC1A activation prevents the differentiation of NSCs into oligodendrocytes by upregulating PTGS2, which leads to increased production and release of PGE2 within extracellular vesicles (EVs). ASIC1A or PTGS2 deficiency in NSCs counters the ASIC1A-related effects on mediating NSC differentiation by reducing PGE2 expression within NSC-derived EVs. Furthermore, intervention in ASIC1A signaling by administration of ASIC1A inhibitors or genetic deletion of ASIC1A demonstrated a pronounced advantage in enhancing myelin sheath regeneration and axonal remyelination.

**Conclusions:**

The activation of ASIC1A prevents NSC differentiation into oligodendrocytes via the transcellular NSC-to-NSC delivery of PGE2, resulting in the failure of myelin sheath regeneration and axonal remyelination following SCI. The inhibition of ASIC1A presents a promising therapeutic strategy for the treatment of SCI.

**Graphical Abstract:**

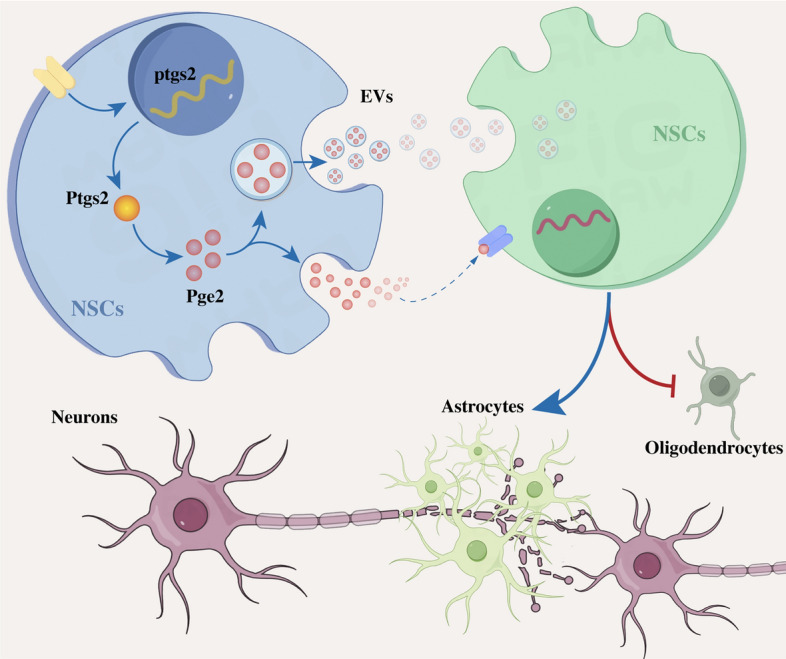

## Introduction

Spinal cord injury (SCI) represents a severe form of neurological trauma resulting from a primary mechanical insult that leads to permanent loss of both motor function and sensory perception [1–3]. This primary injury inflicts direct physical damage to the spinal cord, initiating a complex pathophysiology known as secondary injury. Secondary injury induces production of inhibitory compounds forming an unfavorable microenvironment, which resulted in the progressive loss of surviving nerve cells, demyelination, and death of axons [4–6]. This microenvironmental deterioration represents a significant obstacle to spontaneous recovery after SCI [6–8]. Therefore, understanding the molecular mechanism of the poor microenvironment is critical to finding effective therapies for constricting secondary injury, enhancing spontaneous repair, and improving neurological recovery following SCI.

In the acute-to-subacute phase following SCI, the initial spinal cord insult triggers the excessive generation of adenosine triphosphate (ATP) and glutamate, leading to an augmented production of hydrogen ions (H^+^) [9, 10]. Concurrently, injury-induced vascular damage or dysfunction results in ischemia and hemorrhaging, further contributing to the accumulation of lactic acid. These pathophysiological alterations led to a decrease in pH within the lesion sites, giving rise to acidosis or extracellular acidification, a phenomenon frequently implicated in neurotrauma [11–13]. Acid-sensing ion channels (ASICs) comprise a group of cationic channels that are activated by extracellular H^+^ and belong to the broader family of epithelial sodium channels/degenerin protein superfamily [14–17]. Four genes (*ACCN1, ACCN2, ACCN3, and ACCN4*) encode seven distinct ASIC subunits (ASIC1a, ASIC1b1, ASIC1b2, ASIC2a, ASIC2b, ASIC3, and ASIC4) that have been identified [18]. ASIC1a is widely distributed within the central nervous system (CNS) and becomes activated when extracellular pH falls below 7 [14, 19]. It serves as an acid receptor, transmitting the low pH signal from the extracellular microenvironment into the cell and resulting in substantial influxes of sodium (Na^+^) and calcium (Ca^2+^). The excessive accumulation of these ions, in turn, activate downstream signaling pathways [14, 15]. ASIC1A is activated in nerve cells and immune cells during the pathological progression of neurodegenerative diseases and neurotrauma, including SCI [14, 20–22]. Blockade of ASIC1A could provide neuroprotection and achieved an improvement in neurological recovery following SCI [20, 23]. However, there have been contradicting reports on the role of ASIC1A following SCI, revealing that the activation of ASIC1A could counter acted deleterious effects through raising the excitotoxicity threshold pointing out that ASIC1A might not serve as a viable therapeutic target for SCI [24, 25].

Neural stem cells (NSCs) are capable of self-renew and are multipotent. These cells are present in all main subdivisions of the adult mammalian central nervous system, including the spinal cord. Following SCI, NSCs become active, migrate, and accumulate at lesion sites in an attempt to repair or replace injured nerve cells [26–29]. Due to the poor environment within lesion sites in the early phase of SCI, the majority of these activated endogenous NSCs differentiate into astrocytes rather than oligodendrocytes, resulting in excessive formation of astrocytic scars [26, 30]. However, whether the differentiation of these activated NSCs could be affected by acidosis within lesion sites following SCI through ASIC1A signaling and its molecular mechanism still remains unclear.

In the present study, we elucidate ASIC1A-related signaling pathways through which the acidic environment regulates the differentiation of neural stem cells (NSCs). ASIC1A serves as a pH-drop sensor that is prominently activated within NSCs following SCI. The activation of ASIC1A assumes a pivotal role in inhibiting the differentiation of NSCs into oligodendrocytes. By RNA-sequence analysis, the inhibition of ASIC1A and the knocking down Ptgs2 expression, we unveiled Ptgs2 as a promising downstream target of ASIC1A and the activation of ASIC1A induces significant alterations in the functional properties of extracellular vesicles (EVs) derived from NSCs by enhancing the release of prostaglandin E2 (PGE2). The transcellular NSC-to-NSC delivery of PGE2 inhibited NSCs differentiation into oligodendrocytes, contributing to the failure of myelin sheath regeneration after SCI. By deleting ASIC1A in mice or injecting an ASIC1A inhibitor, we consistently demonstrated that the inhibition of ASIC1A activation enhanced myelin sheath regeneration and axonal remyelination following SCI. Thus, our study found a mechanism by which the acidic microenvironment activates ASIC1A, subsequently facilitating the NSC-mediated transcellular delivery of PGE2. This process exerts a profound influence on the regulation of NSC differentiation, thereby presenting a promising therapeutic target for the treatment of SCI.

## Methods

### Primary NSC culture, differentiation, and transfection

NSC culture was prepared following established protocols detailed in our previous studies [31, 32]. Briefly, NSCs were isolated from the subventricular zone of Sprague‒Dawley rats or C57BL/6 mice and were suspended as neurospheres in NSC culture medium comprising DMEM/F12, 20 ng/mL epidermal growth factor (EGF, Gibco, USA), 2% B27 (Gibco, USA), and 10 ng/mL basic fibroblast growth factor (bFGF, Gibco, USA). To ensure optimal growth, the NSC culture medium was refreshed every 3 days. After 7 days of suspension culture, neurospheres were dissociated into single cells using trypsin and subsequently plated on glass coverslips in NSC differentiation medium containing DMEM/F12, 2% B27 and 1% antibiotic solution. The medium was changed every 3 days. The cells were cultured for 5 days before immunofluorescence staining.

To achieve the knockdown of Asic1a and Ptgs2, small-interfering RNAs (siRNAs) targeting rat Asic1a , Ptgs2 and their respective negative controls were transfected into cells using Lipofectamine ® 2000 reagent (Invitrogen, USA).The target sequences of Asic1a small-interfering were:sense sequenceACGCUUCUCCCUGAAACCUUAdTdT, antisense sequence UAAGGUUUCAGGGAGAAGCGUdTdT. The target sequences of Ptgs2 small-interfering were: sense sequence CCACGGCUGAAGACCAUGAAAdTdT, antisense sequence UUUCAUGGUCUUCAGCCGUGGdTdT. The transfection process was carried out inserum-free Dulbecco’s modified Eagle medium (DMEM), with the siRNAs added at a final concentration of 10 nM according to the instruction of manufacturer. Following transfection, cells were allowed to culture for an additional 24 h in growth media. Subsequently, polymerase chain reaction (PCR) was utilized to validate the expression levels of the target genes.

### NSC-EV collection, identification and labeling

The procedure for NSC-EV collection was described in our previous study with minor modifications [33]. Briefly, primary NSCs were cultured for 6 days with NSC culture medium, and then the medium was replaced with DMEM/F12. Following an additional 24 h incubation, the medium was collected as conditioned medium. To eliminate cell debris, the collected conditioned medium underwent gradient centrifugation at 4 °C. Following a 90 min centrifugation at 100,000*g* at 4 °C, NSC-EVs were harvested and resuspended in 100 μL of PBS (Gibco) before being stored at −80 °C. The concentration of NSC-EVs was detected by a BCA protein assay. Dynamic light scattering and TEM were performed to detect the diameters and analyze the morphology of NSC-EVs. Western blotting was used to detect CD63 (1:1000), CD9 (1:1000), and TSG101 (1:1000) expression within NSC-EVs. An ELISA kit (ELK Biotechnology, China) was used to test the concentration of Pge2 within NSC-EVs.

Pkh-26 (Sigma, Germany) was used to label NSC-EVs according to the manufacturer’s protocol. Briefly, 4 μL Pkh-26 was diluted in 100 μL diluent C solution before being incubated for 10 min at room temperature with the resuspended NSC-EVs. To recollect the NSC-EVs, samples were centrifuged at 100,000*g* for 90 min at 4 °C. The collected Pkh-26-labeled NSC-EVs were washed twice with DMEM/F12 medium to eliminate the free red fluorescent membrane linker dye.

### An animal spinal cord injury model, intrathecal injection and behavioral test

Adult (6–8 weeks) female SD rats and C57BL/6 mice from the Animal Facility of Anhui Medical University and Asic1a-knockout (KO) mice from Shanghai Model Organisms Center were used to induce a severe crush SCI model according to a previous study [34]. Animals were housed in clean and warm cages at an ambient temperature of 22–25 °C on a 12 h light/dark cycle with an adequate supply of food and water. Animal procedures were approved by the Animal Ethics Committee of Anhui Medical University in accordance with the Basel Declaration (approval no. LLSC 20210675; date: March 2021). All operations were performed to minimize the number of animals used and to reduce animal suffering. Animals were blindly assigned to treatment groups and anesthetized. A laminectomy was performed at T10 to expose the spinal cord before performing a crush by using Dumont forceps with a 0.5 mm tip to completely compress the spinal cord from both lateral sides for 5 s. The intrathecal injection site was performed at the dorsal midpoint of the lumbar 5–6 intervertebral space. A catheter was placed under the dura and connected to a mini-osmotic pump (Alzet 1007D, USA) filled with normal saline (served as SCI groups), the nonspecific ASIC antagonist amiloride (MCE, USA), or the specific ASIC1a antagonist psalmotoxin 1 (MCE, USA). The pump was placed under the back skin and removed following a 3-day continuous intrathecal injection. To detect tissue pH, a laminectomy was performed to expose lesion site (1 cm centered on the epicenter of the compressed lesion) and adjacent segment (1 cm from the cranial edge of lesion site). A micro-pH meter (Thermo Fisher Scientific, USA) was inserted and recorded pH value into these two regions. The locomotor functional deficits were measured blindly by using the Basso, Beattie, and Bresnahan (BBB) Locomotor Rating Scale and the inclined plane test every week. After the accomplishment of the necessary assessments, all rats were euthanized using carbon dioxide.

### Immunofluorescence staining

Cells fixation was performed using 4% paraformaldehyde for 30 min. For tissue processing, animals were fixed by transcardiac perfusion with ice-cold phosphate-buffered saline followed by 4% paraformaldehyde. Spinal cords were harvested and fixed in 4% PFA at 4 °C for 24 h, then spinal cords were sectioned into 4-μm thick longitudinal slices, focusing on the core of the damaged lesion. A Leica RM2135 electric slicer (Leica, Germany) was utilized for this purpose. The primary antibodies were incubated overnight at 4 °C for immunofluorescence staining as follows: rabbit anti-glial fibrillary acidic protein (GFAP) for astroglia (1:1000; Abcam, United Kingdom), mouse anti-CNPase for oligodendrocytes (1:200; Abcam, United Kingdom), rabbit anti-myelin basic protein (MBP) for oligodendrocytes (1:1000; Abcam, United Kingdom), rabbit anti-neuron-specific class III beta-tubulin (Tuj1) for neurons (1:1000; Abcam, United Kingdom), mouse anti-Asic1a (1:200; Abcam, United Kingdom), mouse anti-prostaglandin-endoperoxide synthase 2 (Ptgs2; 1:200; Santa Cruz, USA), and rabbit anti-prostaglandin E2 receptor EP2 subtype (PTGER2). The secondary antibodies were Alexa Fluor 488 (green, 1:50; Elabscience, China) and Cy3 (red, 1:50; Elabscience, China). DAPI was used for nuclear staining. Observation and image capture of stained tissue slices were conducted using a DM-6B fluorescence microscope (Leica, Germany). The data were collected by ImageJ. To count the number of positive cells in vitro, samples were collected from five individuals. For each culture condition, we randomly selected 10–15 fields with a total of 500–1000 cells for quantification of positive cells. To quantify the percentage of positive cells in vivo, five individual animals were selected in every group and in each individual and three 4-μm-thick longitudinal slices were obtained from each animal. We selected 3–5 random fields in each slice for quantification of positive cells.

### Western blot assay

To extract proteins, cells, or a 0.5 cm segment of the damaged spinal cord, centered around the epicenter of the injured lesion, were lysed in lysis buffer. The concentration of the collected protein was determined using a BCA protein assay kit. Equal amounts (20 μg) of protein were loaded and separated by sodium dodecyl sulfate polyacrylamide gel electrophoresis (SDS‒PAGE) before being transferred to a PVDF membrane, which was blocked with 5% skim milk for 1 h. Subsequently, the membranes were incubated with primary antibodies (ASIC1A, 1:1000, Abcam, United Kingdom) overnight at 4 °C before being incubated with secondary antibodies (1:5000; Elabscience, China) at room temperature for 1 h. The protein bands were visualized using the Super-Signal West Pico enhanced chemiluminescence reagent (Advansta, USA). ImageJ software was used to perform protein analysis.

### RNA extraction, quantitative polymerase chain reaction (PCR) and RNA-sequencing

RNA extraction from cells was performed using TRIzol reagent (Gibco, USA) in accordance with the manufacturer's instructions. Subsequently, cDNA was synthesized using Superscript III RT Reaction Mix (Invitrogen). Quantitative PCR was conducted using a RealPlex2 Mastercycler (Eppendorf) and SYBR Green Master Mix (Applied Biosystems). GAPDH was used as the reference gene for normalizing mRNA expression. The primer sequences for the specific transcripts were as follows: ptgs2, 5′ACAACAACTCCATCCTCCTTGAA-3′, 5′-TCATCTCTCTGCTCTGGTCAATG -3′; gapdh, 5′- CAAGGCTGAGAATGGGAAGC-3′, 5′-GAAGACGCCAGTAGACTCCA-3.

For RNA-sequence analysis, RNA was extracted from NSCs cultured in normal NSC differentiation medium or acidic NSC differentiation medium at pH 6.0 for 24 h. RNA sequencing and data analysis were performed by Biomarker Technologies Corporation (Beijing, China). The experimental procedure of mRNA sequencing included sample preparation, library construction, library quality control, and sequencing. The qualified cDNA library was sequenced on an Illumina sequencing platform.

### TUNEL staining

Cells were seeded at a density of 1 × 10^5^ per well and incubated with normal medium or pH 6.0 acidic medium for 24 h. A TUNEL test kit (Beyotime, China) was used to calculate apoptotic rates following the instructions provided by the manufacturer.

### Summary of animal groups

**Fig. 1:**
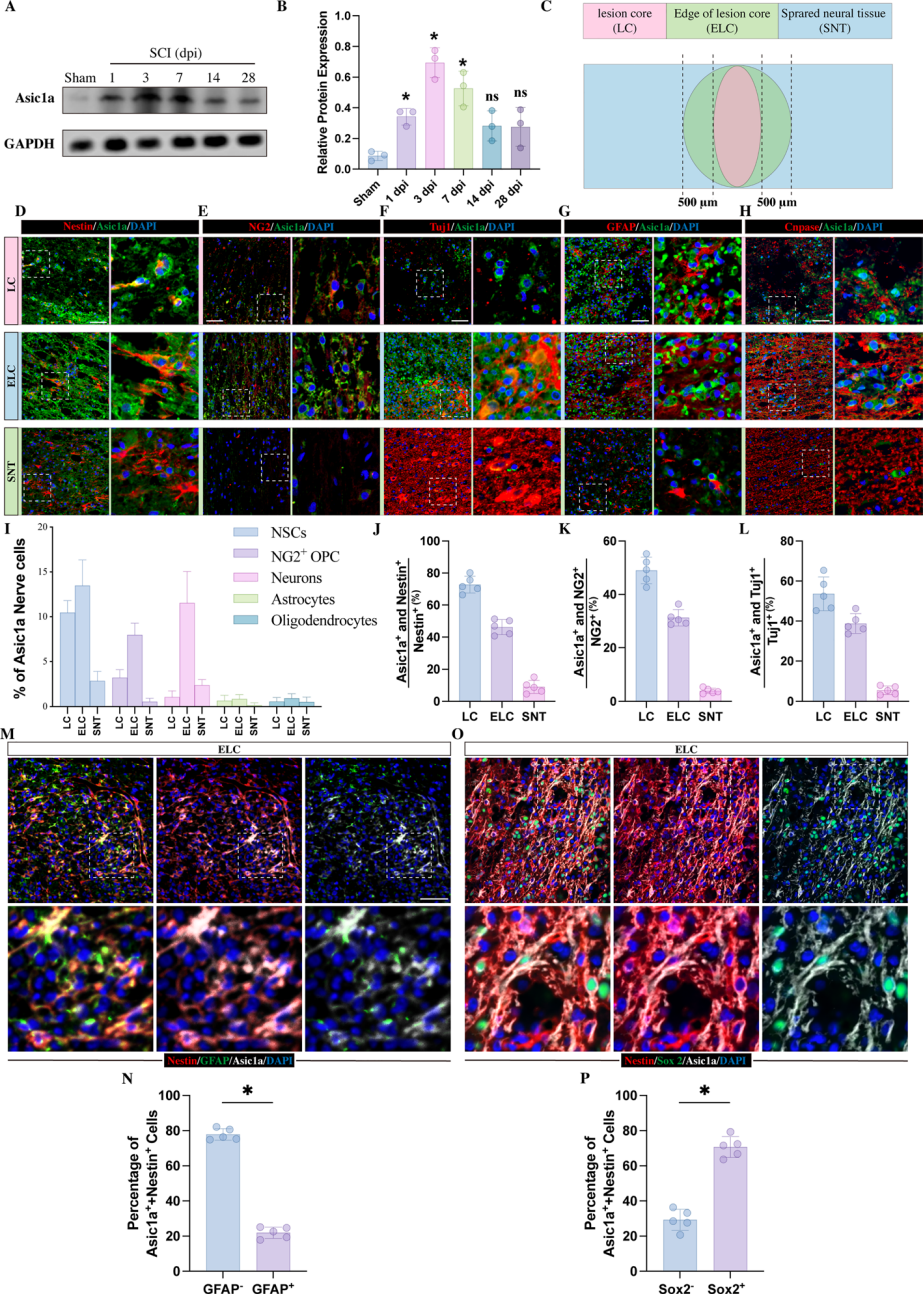
ASIC1A was upregulated in NSCs following SC. **A**, **B** Western blot analysis of ASIC1A in the lesion sites at different time points following SCI (*n* = 3, *p < 0.05 compared with Sham groups, ^ns^*p* > 0.05 compared with Sham groups). **C** Experimental summary schematic showing the view of the lesion core (LC), edge of the lesion core (ELC), and spread neural tissue (SNT) following SCI. **D**–**H** Immunofluorescence staining of ASIC1A (green) with NESTIN (red, **D**), NG2 (red, **E**), Tuj1 (red, **F**), GFAP (red, **G**), or Cnpase (red, **H**) in the LC, ELC, and SNT on day 3 post-injury. Scale bar, 50 μm. **I** Quantitation of the percentage of NESTIN^+^, NG2^+^, TUJ1^+^, GFAP^+^, and CNPASE^+^ cells co-expressing ASIC1A (*n* = 5). **J** Quantitative analysis of the percentage of ASIC1A and NESTIN double-positive cells among the total NESTIN-positive cells (*n* = 5). **K** Quantitative analysis of the percentage of ASIC1A and NG2 double-positive cells among the total NG2-positive cells (*n* = 5).** L** Quantitative analysis of the percentage of ASIC1A and TUJ1 double-positive cells relative to the total TUJ1-positive cells (*n* = 5). **M**, **N** Immunofluorescence staining of ASIC1A with NESTIN and GFAP in the ELC at 3 dpi. Scale bar, 50 μm. **O**, **P** Immunofluorescence staining of ASIC1A with NESTIN and Sox 2 in the ELC at 3 dpi. Scale bar, 50 μm. All data are presented as the mean ± standard deviation (SD)

**Fig. 2:**
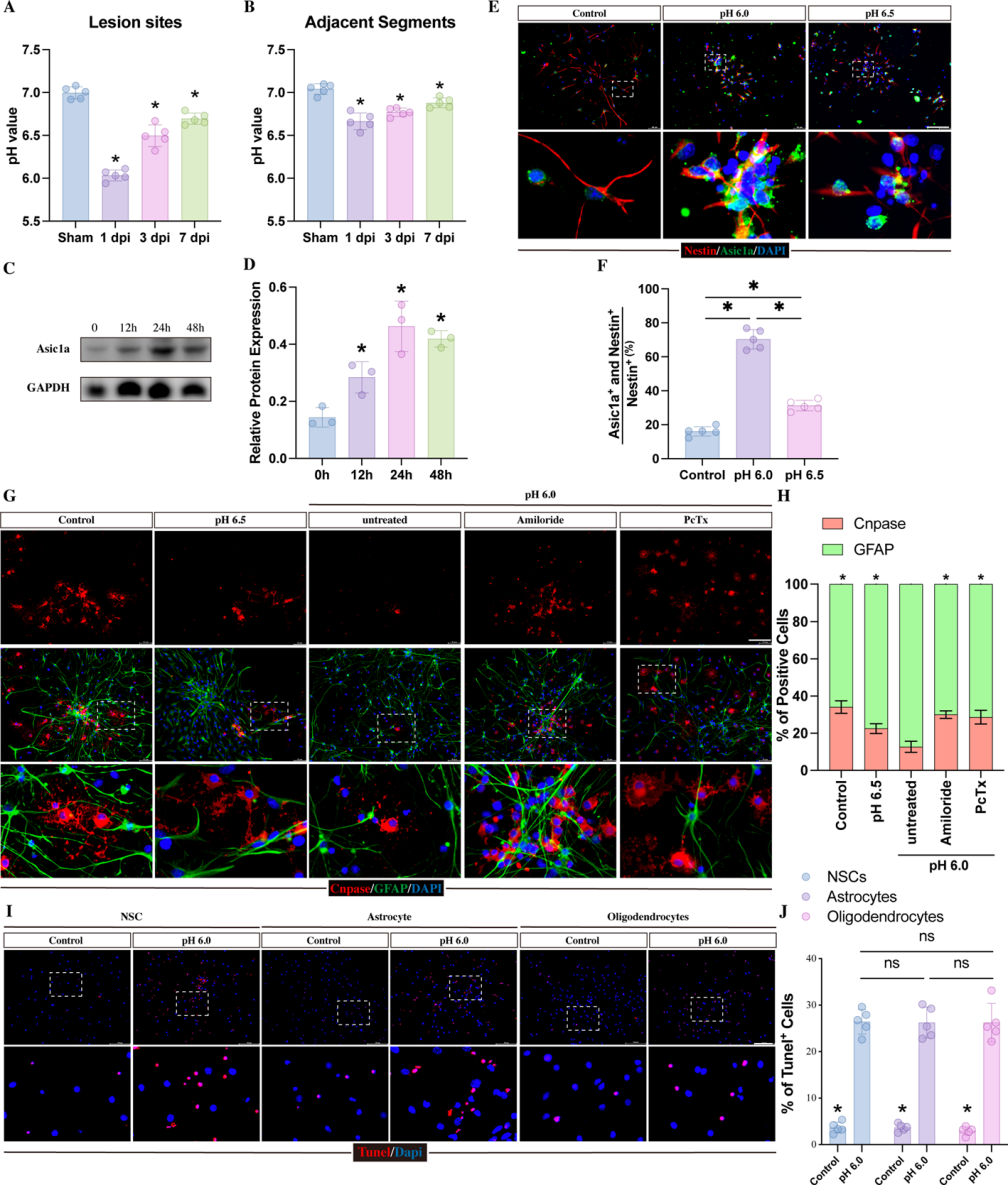
Acidic medium-induced activation of ASIC1A inhibits NSC differentiation into oligodendrocytes. **A**, **B** Tissue pH was directly detected in lesion site and adjacent segment by a micro–pH meter at different time points following SCI (*n* = 5). **C**, **D** Western blot analysis of ASIC1A in NSCs following 12 h, 24 h, and 48 h of acidic medium treatment (*n* = 3). **E**, **F** Immunofluorescence staining of ASIC1A (green) and NESTIN (red) in NSCs treated with normal medium (control groups) and acidic medium (pH 6.0 groups or pH 6.5 groups) for 24 h (*n* = 5; Scale bar, 100 μm). **G** Representative images of GFAP (green) and CNPASE (red) immunofluorescence staining after 5 days of treatment with normal medium treatment, acidic medium treatment, and acidic medium treatment with 100 μM amiloride or 30 nM PcTx. Scale bar, 100 μm. **H** Quantitation of the percentage of CNPASE-positive oligodendrocytes and GFAP-positive astrocytes from the experiments shown in **E** (*n* = 5, each treatment was compared with the pH 6.0 groups). **I**, **J** TUNEL staining revealing the apoptosis of NSCs, astrocytes and oligodendrocytes cultured with normal medium or pH 6.0 acidic medium for 24 h (*n* = 5; scale bar, 100 μm). All data are presented as the mean ± SD; *p < 0.05

**Fig. 3:**
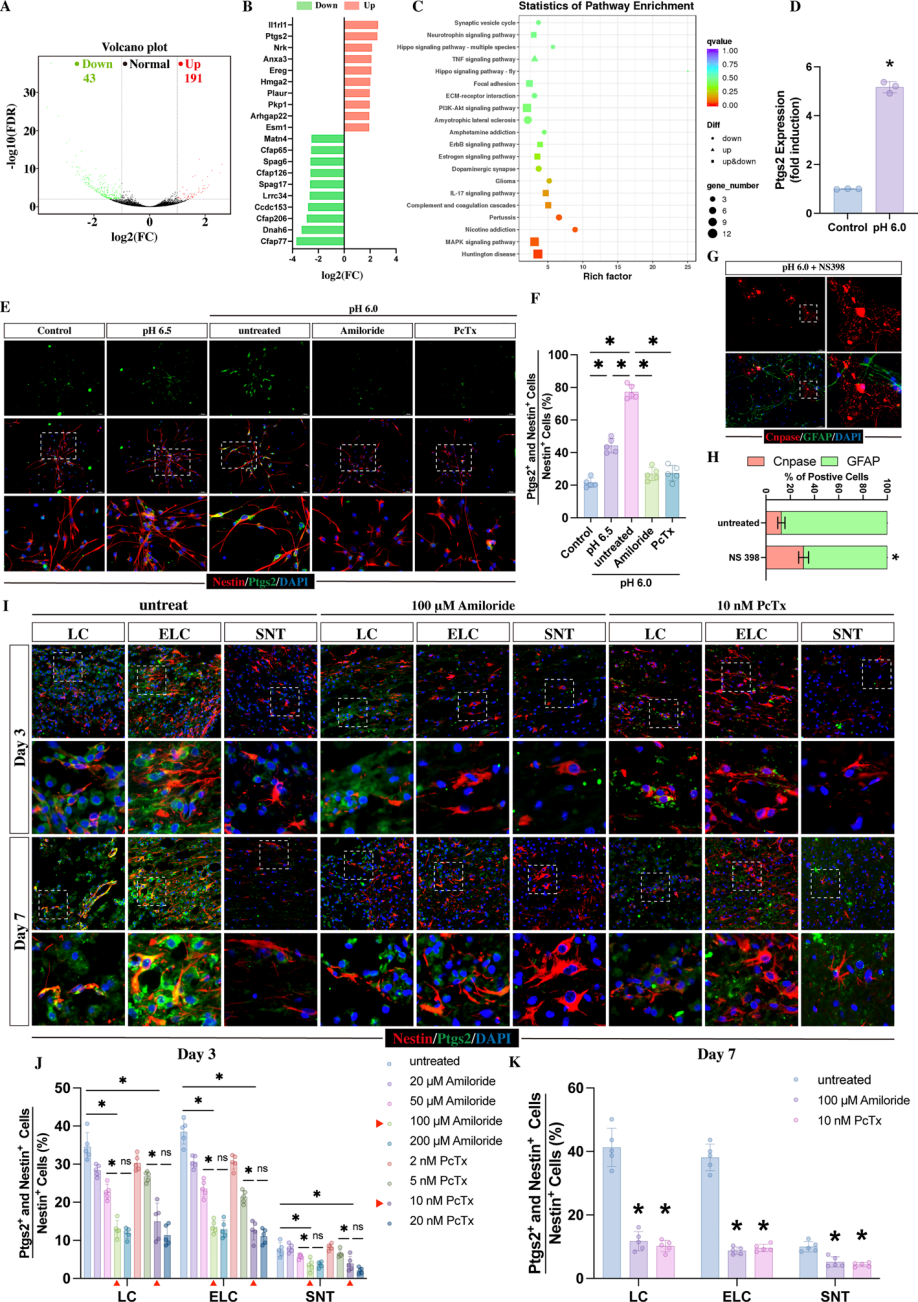
Acidic medium treatment upregulates Ptgs2 expression via ASIC1A activation in NSCs. **A** Volcano plot showing the number of genes that were altered by 24 h of treatment with acidic medium. **B** The top ten upregulated and downregulated genes (ranked by log_2_FC). **C** Statistics of pathway enrichment **D** Quantitative real-time (RT)‒PCR analysis of the relative expression of *ptgs2* genes after 24 h acidic medium treatment normalized to controls. **E** Immunofluorescence staining of PTGS2 (green) and NESTIN (red) in NSCs treated with normal medium, acidic medium, or acidic medium with 100 μM amiloride or 30 nM PcTx for 24 h. Scale bar, 100 μm. **F** Quantitative analysis of the percentage of Ptgs2 and Nestin double-positive cells relative to the total NESTIN-positive cells in each treatment (*n* = 5). **G**, **H** Representative images of GFAP (green) staining and CNPASE (red) immunofluorescence staining after 5 days of acidic medium culture containing 50 μM NS398 (Ptgs2 inhibitor) (*n* = 5, *p < 0.05 compared with NSCs that received acidic medium treatment at pH 6.0; Scale bar, 100 μm). **I** Immunofluorescence staining of PTGS2 with NESTIN in the LC, ELC, and SNT at days 3 and 7 post-injury in rats that received the treatment of amiloride or PcTx. Scale bar, 50 μm. **J** Quantitation of the percentage of PTGS2 and NESTIN double-positive cells relative to the total NESTIN-positive cells at 3 dpi in rats that received the treatment of different concentration of amiloride or PcTx (*n* = 5, *p < 0.05 compared with the untreated SCI rats). **K** Quantitation of the percentage of PTGS2 and NESTIN double-positive cells relative to the total NESTIN-positive cells at 7 dpi (*n* = 5, *p < 0.05 compared with the untreated SCI rats). All data are presented as the mean ± SD

**Fig. 4:**
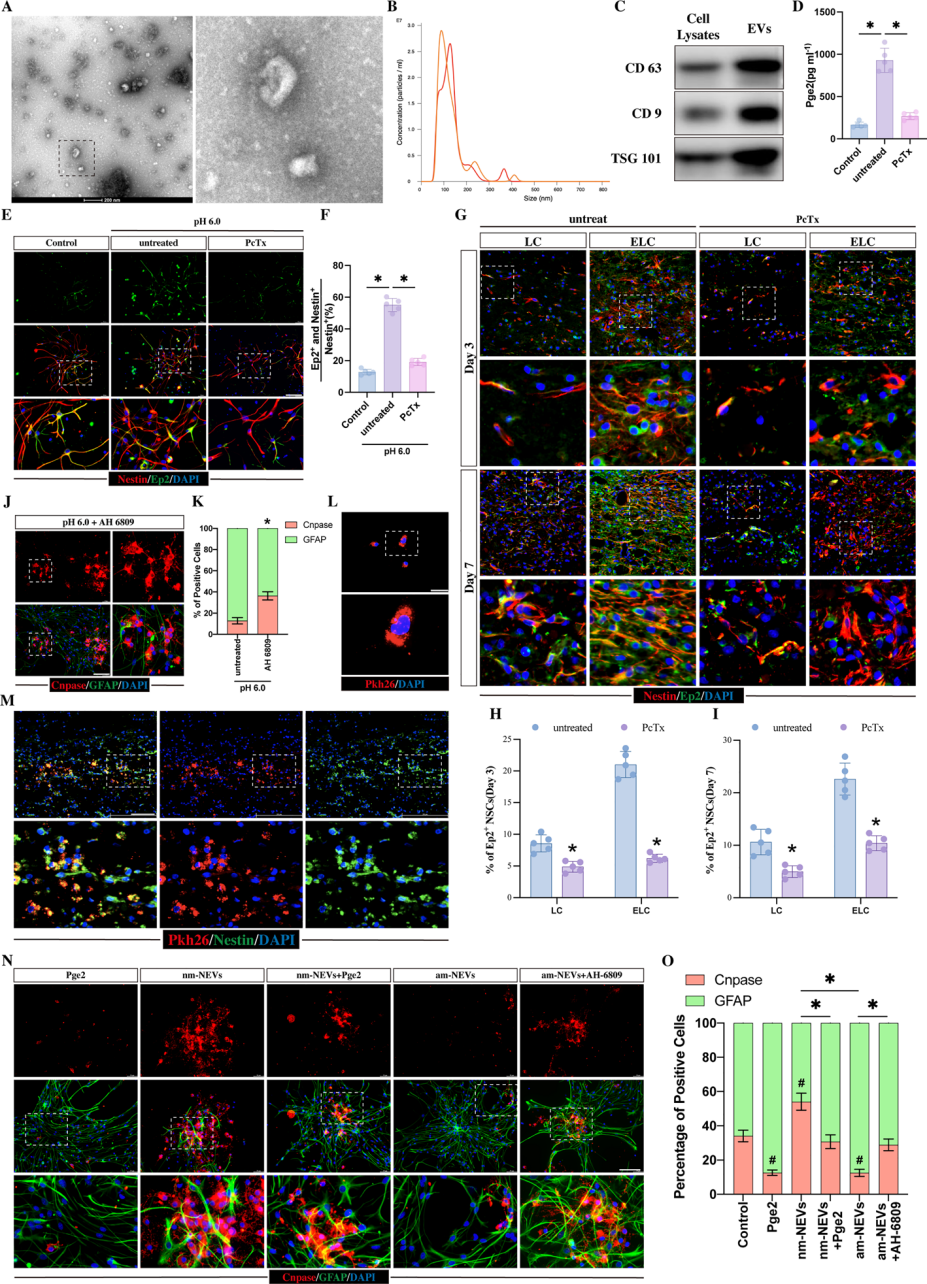
Activation of ASIC1A inhibit NSC differentiation into oligodendrocytes by trans-cellular derivery of PGE2. **A**–**C** NSC-EV identification through TEM (**A**), dynamic light scattering (**B**), and western blot analysis (**C**).** D** ELISA kit measurement of PGE2 concentration in EVs derived from NSCs receiving either normal medium (control groups) or acidic medium (pH 6.0 untreated groups) with or without 30 nM PcTx (*n* = 5, *p < 0.05). **E** EP2 and NESTIN co-immunofluorescence staining of NSCs treated with normal medium or acidic medium with or without 30 nM PcTx for 24 h. Scale bar, 100 μm. **F** Quantitative analysis of the percentage of EP2 and NESTIN double-positive cells among NESTIN-positive NSCs in each treatment (*n* = 5, *p < 0.05). **G** Immunofluorescence staining of EP2 with NESTIN in LC and ELC and SNT at days 3 and 7 post-injury from SCI rats with or without 10 nM PcTx. Scale bar, 50 μm. **H**, **I** Quantitation of the percentage of EP2 and NESTIN double-positive cells on day 3 (**H**) and Day 7 post-injury (**I**) (*n* = 5; *p < 0.05 compared with the untreated SCI rats). **J**, **K** Representative images of GFAP (green) and CNPASE (red) immunofluorescence staining after 5 days of acidic medium culture with 1 μM AH 6809 (a prostaglandin E2 receptor antagonist) (*n* = 5, *p < 0.05 compared with NSCs that received acidic medium treatment at pH 6.0; Scale bar, 100 μm). **L** Pkh-26-labeled EVs were found within the cytoplasm of NSCs after 24 h of coculture. Scale bars, 50 μm. **M** Intrathecal injected Pkh-26-labeled EVs were taken up by NESTIN-positive NSCs in the injured lesion sites on day 3 post-injury. Scale bar, 100 μm. **N** Representative images of GFAP (green) and CNPASE (red) immunofluorescence staining of NSCs that received 5 days of treatment with normal medium (served as a control) with or without 10 μM Pge2, normal medium containing 25 μL nm-NEVs with or without 10 μM PGE2, and normal medium containing 25 μL am-NEVs with or without 1 μM AH 6809 (scale bar, 100 μm; nm-NEVs are abbreviated for EVs derived from normal medium-treated NSCs; am-NEVS is abbreviated for EVs derived from acidic medium-treated NSCs). **O** Quantitation of the percentage of CNPASE-positive oligodendrocytes and GFAP-positive astrocytes from the experiments shown in **E** (*n* = 5; *p < 0.05; ^#^*p* < 0.05 compared with control groups). All data are presented as the mean ± SD

**Fig. 5:**
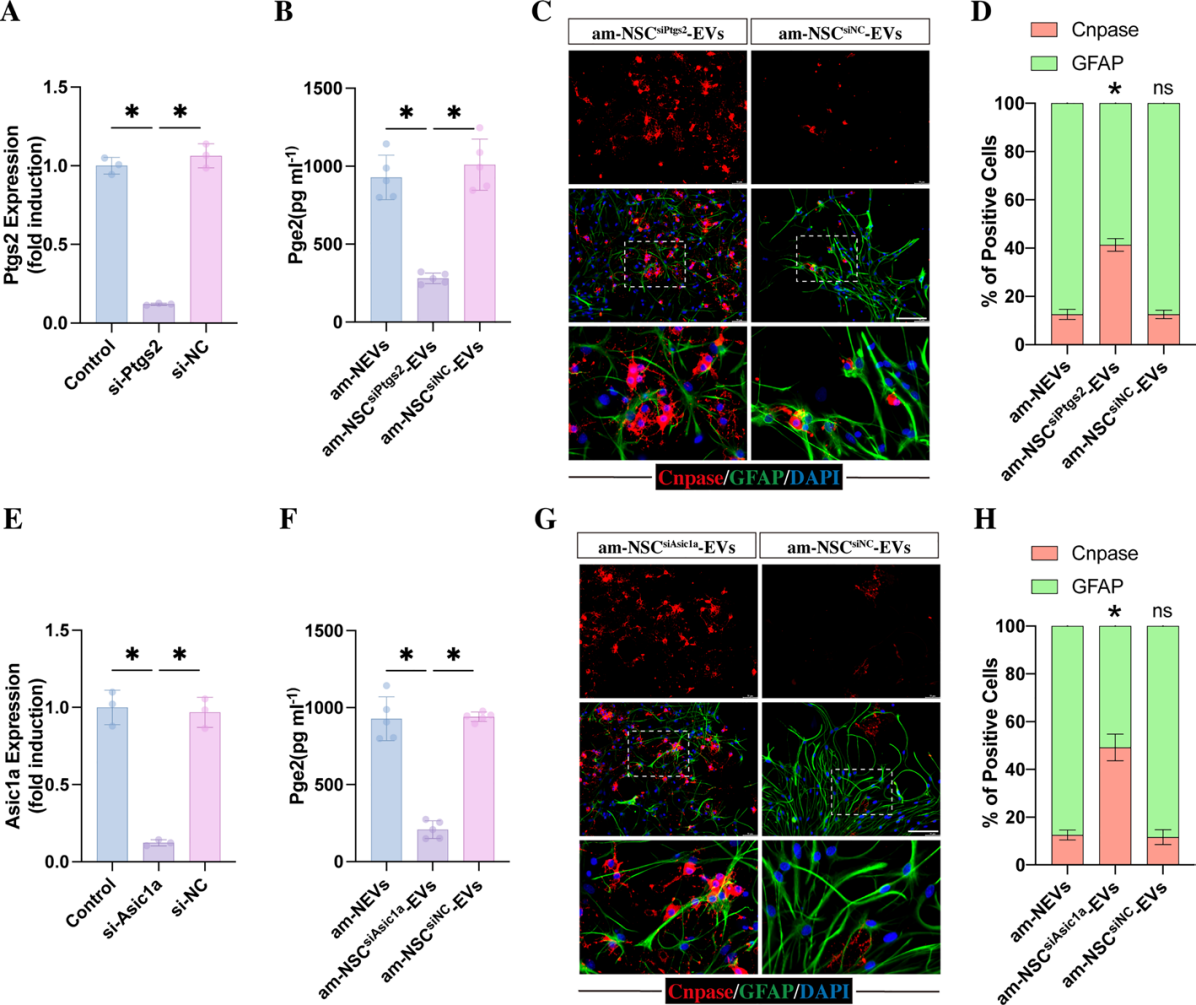
EVs derived from Asic1a- or Ptgs2-Deficient NSCs induced NSC differentiation into oligodendrocytes. **A** Quantitative RT‒PCR analysis of the relative expression of *ptgs2* genes in si-Ptgs2-transfected NSCs, confirming the knockdown of *ptgs2* gene expression (*n* = 3, normalized to controls, *p < 0.05). **B** ELISA was used to detect the concentration of PGE2 in EVs derived from acidic medium-treated NSCs, si-Ptgs2-transfected NSCs and si-NC-transfected NSCs (*n* = 5, *p < 0.05). **C** Representative images of GFAP (green) and CNPASE (red) immunofluorescence staining of NSCs that received 5 days of treatment with normal medium containing 25 μL EVs derived from acidic medium-treated NSCs, acidic medium-treated and si-Ptgs2-transfected NSCs, and acidic medium-treated and si-NC-transfected NSCs (scale bar, 100 μm). **D** Quantitation of the percentage of CNPASE-positive oligodendrocytes and GFAP-positive astrocytes from the experiments shown in **C** (*n* = 5; *p < 0.05; ^ns^*p* > 0.05 compared with the am-NEV group). **E** Quantitative RT‒PCR analysis of the relative expression of Asic1a genes in si-Asic1a-transfected NSCs, confirming the knockdown of *asic1a* gene expression (*n* = 3, normalized to controls, *p < 0.05). **F** ELISA was used to detect the concentration of PGE2 in EVs derived from acidic medium-treated NSCs, si-Asic1a-transfected NSCs and si-NC-transfected NSCs (*n* = 5, *p < 0.05). **G** Representative images of GFAP (green) and CNPASE (red) immunofluorescence staining of NSCs that received 5 days of treatment with normal medium containing 25 μL EVs derived from acidic medium-treated NSCs, acidic medium-treated and si-Asic1a-transfected NSCs, and acidic medium-treated and si-NC-transfected NSCs (scale bar, 100 μm). **H** Quantitation of the percentage of CNPASE-positive oligodendrocytes and GFAP-positive astrocytes from the experiments shown in **C** (*n* = 5; *p < 0.05; ^ns^*p* > 0.05 compared with the am-NEV group). All data are presented as the mean ± SD

**Fig. 6:**
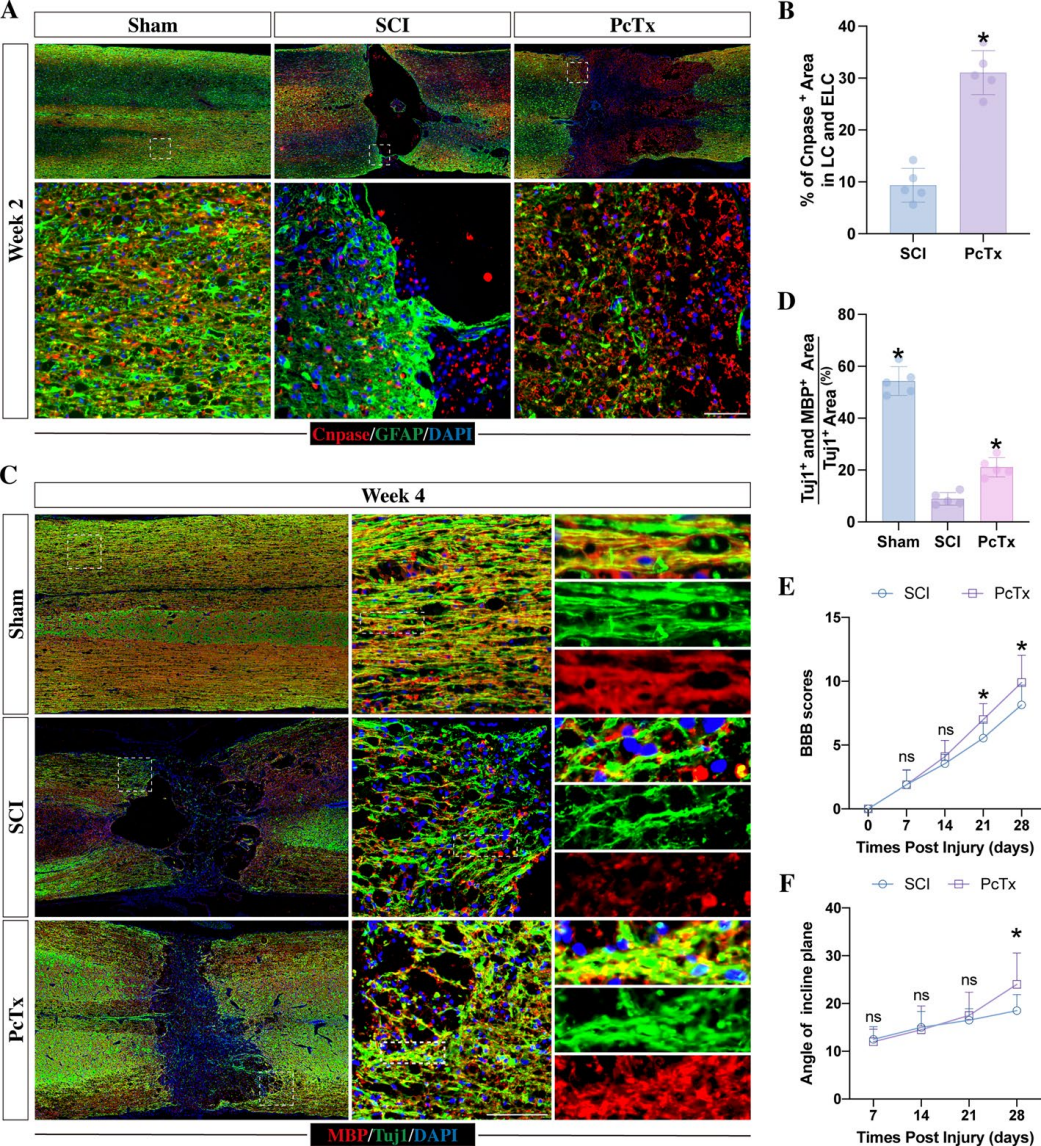
Inhibition of ASIC1A promoted myelin sheath regeneration and axonal remyelination following SCI. **A** Immunofluorescence staining of GFAP and Cnpase at week 2 post-injury in the rats that received or did not receive the injection of PcTx. Scale bar, 100 μm. **B** Quantitation of the percentage of CNPASE-positive areas in the LC and ELC (*n* = 5; *p < 0.05 compared with the SCI rats that only received saline treatment). **C** Immunofluorescence staining of MBP and TUJ1 at week 4 post-injury in the rats that received or did not receive the injection of PcTx. Scale bar, 100 μm. **D** Quantitation of the percentage of MBP- and TUJ1-double positive areas to TUJ1-positive areas in the corticospinal tract of LC and ELC (*n* = 5; *p < 0.05 compared with the SCI rats that only received saline treatment). **E**, **F** The assessment of neurological functional recovery at different time points following SCI by BBB scores and the inclined plane test (*n* = 10, *p < 0.05; ^ns^*p* > 0.05). All data are presented as the mean ± SD

**Fig. 7:**
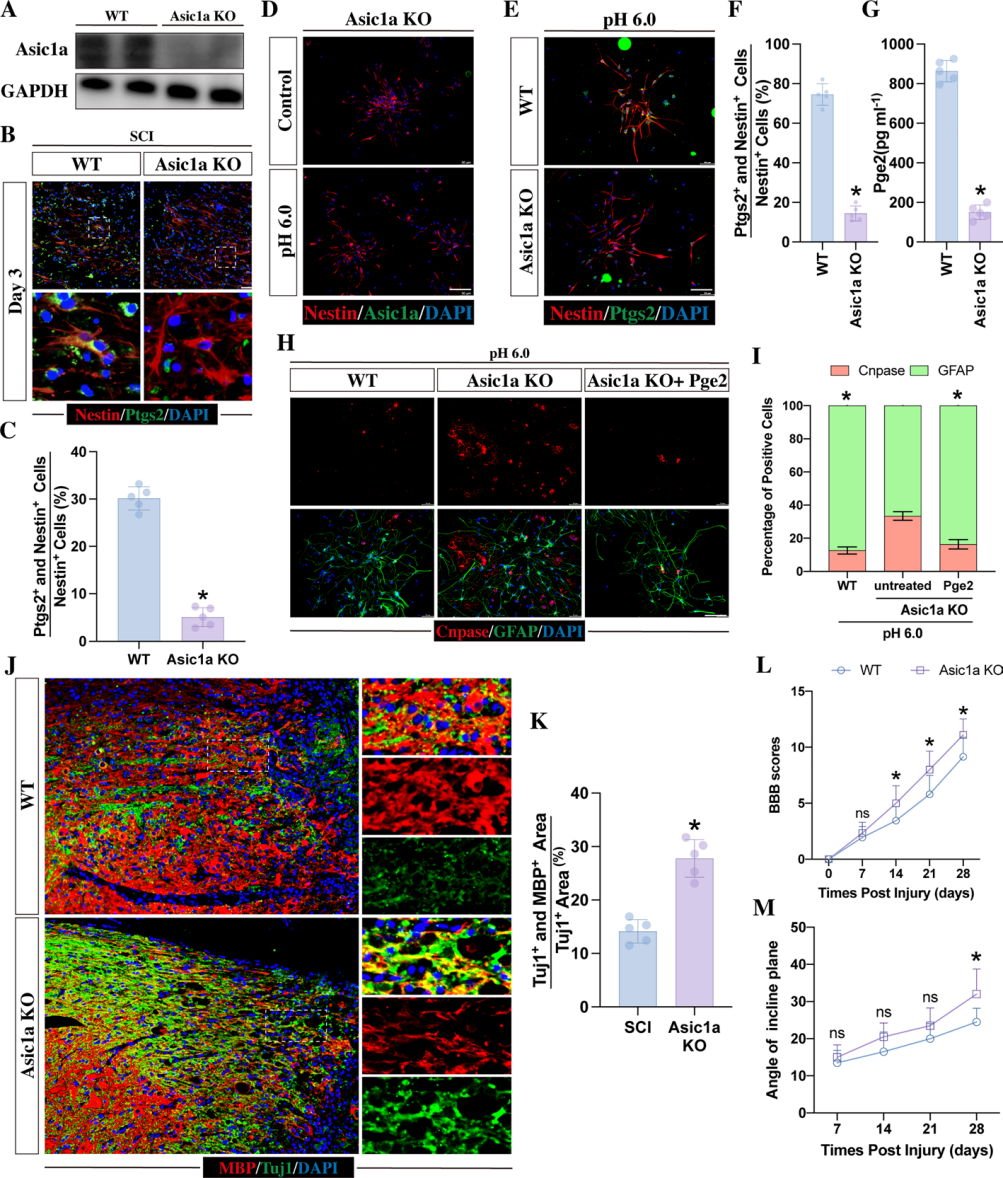
Asic1a-KO in mice increases axonal remyelination via the mediation of NSC differentiation by regulating PTGS2. **A** Western blot analysis of ASIC1A in the spinal cord of WT or Asic1a-KO mice confirming the complete loss of Asic1a protein in Asic1a-KO mice. **B**, **C** Ptgs2 and Nestin immunostaining in WT and Asic1a-KO SCI mice and quantitation of the percentage of double-positive cells in the LC and ELC on day 3 post-injury (scale bar, 100 μm; *n* = 5, *p < 0.05). **D** Immunofluorescence staining of ASIC1A and NESTIN in NSCs from WT and Asic1a-KO mice. **E**, **F** Immunofluorescence staining of PTGS2 and NESTIN in NSCs from WT and Asic1a-KO mice and quantitation of the percentage of double-positive cells after 5 days of acidic medium culture at pH 6.0 (scale bar, 100 μm; *n* = 5, *p < 0.05). **G** ELISA was used to detect the concentration of PGE2 in EVs derived from WT or Asic1a-KO NSCs stimulated with acidic medium (*n* = 5, *p < 0.05). **H**, **I** GFAP and CNPASE immunofluorescence staining of NSCs from WT and Asic1a-KO mice after 5 days of treatment with acidic medium with or without Pge2 addition (scale bar, 100 μm; *n* = 5, *p < 0.05 compared with Asic1a-KO NSCs). **J** Immunofluorescence staining of MBP and TUJ1 at week 4 post-injury in WT and Asic1a mice. Scale bar, 100 μm. **K** Quantitation of the percentage of MBP- and TUJ1-double-positive areas in the corticospinal tract of the LC and ELC (*n* = 5; *p < 0.05). **L**, **M** The assessment of neurological functional recovery by BBB scores and the inclined plane test (*n* = 10, *p < 0.05; ^ns^*p* > 0.05). All data are presented as the mean ± SD. All data are presented as the mean ± SD

The experiments of western blot described in Fig. 1A, B included a total of 18 rats dividing into six groups. In Fig. 1D–P, five rats subjected to SCI and analyzed by immunostaining. In Fig. 2A, B, 10 rats were divided into sham groups; a total of 30 rats subjected to SCI were divided into 3 experimental groups. In Fig. 2C–H, NSCs were obtained from five rats were utilized for immunostaining and western blot examination. In Fig. 2I, J, NSCs, astrocytes and oligodendrocytes used for TUNEL staining were obtained from a total of 15 rats. The experiments of RNA-sequence revealed in Fig. 3A–C included a total of six rats, NSCs cultured from three rats were treated with normal medium and served as control groups, and NSCs obtained from the other three rats were treated with pH 6.0 acidic medium and served as pH 6.0 groups. In Fig. 3D, NSCs were obtained from a total of six rats and were assigned to the control group and the pH 6.0 group. In Fig. 3E–H and Fig. 4E, F, and Fig. 4J, K, NSCs were obtained from a total of five rats and divided into seven groups for immunostaining. In Fig. 3I, J, five rats each were assigned to four amiloride-treated groups and four PcTx-treated groups and were performed immunostaining at 3 dpi. In Fig. 3I, K, a total of ten rats were equally divided into amiloride-treated and four PcTx-treated groups. The data from Fig. 4M included three rats. In Fig. 4N, O, a total of 25 rats were divided into 5 experimental groups. In Fig. 5A, E, NSCs obtained from three rats and divided into three groups. In Fig. 5B, F, NSCs obtained from five rats and divided into three groups. In Fig. 5C, D and Fig. 5G, H, NSCs were obtained from five rats and divided into four groups. In Fig. 6A, B, a total of 15 rats were divided into 3 groups and performed immunostaining following 2 weeks post injury. In Fig. 6E, F, sample sizes of behavioral assessment were determined based on power estimates. A total of 30 rats were assigned to three groups and were assessed for locomotor functional recovery. Among these rats, in each group, five rats were performed immunostaining following 4 weeks post injury, revealing in Fig. 6C, D. In Fig. 7A, two wild-type (WT) mice and 2 Asic1a-KO mice were analyzed for western blotting. The data revealed in Fig. 7B, C included five WT and five Asic1a-KO mice. In Fig. 7D, immunostaining was performed on NSCs obtained from additional two WT mice and two Asic1a-KO mice. The data revealed in Fig. 7E, F included five WT and five Asic1a-KO mice. In Fig. 7G–I, NSCs obtained from five WT and five Asic1a-KO mice were analyzed for immunostaining and ELISA. In Fig. 7J–M, ten WT and ten Asic1a-KO mice were assessed for locomotor functional recovery. Among these mice, in each group, five mice were performed immunostaining following 4 weeks post injury.

### Statistical analysis

All data are presented as the mean ± standard deviation (SD). Statistical analysis was performed using Prism 8.0. The significance of the data was assessed using Student's *t*-test for comparisons involving two groups, and one-way analysis of variance (ANOVA) was employed for analyses involving more than two groups, followed by Tukey’s post hoc procedure for multiple comparisons. Mann–Whitney test and Kruskal–Wallis test were used for non-parametric data. The threshold for determining statistical significance was set at *p* < 0.05.

## Results

### ASIC1A was upregulated in NSCs following SCI

To address whether ASIC1A was upregulated following SCI, we detected the expression of ASIC1A within the lesion sites at different time points post-injury by western blot. The expression level of ASIC1A was increased at 1 day post-injury (dpi), reached its peak at 3 dpi, and then gradually decreased (Fig. 1A, B). To further investigate which type of nerve cells expressed ASIC1A, we co-labeled ASIC1A with different nerve cells by immunostaining in SCI rats. Given that the highest expression of ASIC1A was observed at 3 dpi, we focused on this time point for immunostaining. Based on the characteristics of cell biology following SCI, the injured spinal cord was divided into three regions: the lesion core (LC), the edge of the lesion core (ELC), and the surrounding neural tissue (SNT) (Fig. 1C). Compared with the SNT, which was remote from the LC, the ASIC1A expression level was strongly increased in the LC and ELC regions. In these two regions, ASIC1A was found to be colocalized with NSCs, NG2-positive oligodendrocyte precursor cells (OPCs), and Tuj-1-positive neurons (Fig. 1D–F). In the LC, 10% of ASIC1A and Nestin double-positive NSCs (accounting for 72.8% of total NSCs, Fig. 1D, I, J) and 3.2% of ASIC1A and NG2 double-positive OPCs (accounting for 48.9% of total OPCs) were found in this region (Fig. 1E, I, K). Although 56% of TUJ1-positive neurons (Fig. 1L) were found to express ASIC1A, due to the initial damage-induced loss of neurons, only a few neurons were found in this region, resulting in an extremely low percentage of TUJ1- and ASIC1A-double-positive cells (Fig. 1F, I). In the ELC, ASIC1A was also found to be colocalized mainly with NSCs, OPCs and TUJ-1-positive neurons. The double-positive percentage was 13.4% in NSCs (accounting for 46.2% of total NSCs, Fig. 1D, I, J) and 8% in NG2^+^ OPCs (accounting for 38.7% of total OPCs, Fig. 1E, I, K). With an increased number of TUJ1-positive neurons in the ELC, the percentage of TUJ 1- and ASIC1A-double-positive cells rose to 11.5% (Fig. 1F, I), accounting for 38.8% of the total neurons (Fig. 1L). In the SNT, where ASIC1A expression was considerably reduced, there was a corresponding decrease in the percentage of ASIC1A-positive NSCs, OPCs and neurons (Fig. 1D–F, I–L). In contrast, in these three regions, few GFAP-positive astrocytes and CNPASE-positive oligodendrocytes co-expressed ASIC1A (Fig. 1G–I).

Although Nestin was highly expressed in NSCs, it was not a specific marker to identify NSCs. It has been reported to be expressed in reactive astrocytes [35]. To confirm whether these Nestin-positive cells were from reactive astrocytes, we co-labeled ASIC1A with Nestin and GFAP by immunostaining in ELC at 3 dpi. The results showed that among the Nestin- and ASIC1A-double positive cells, only 20% cells were co-labeled with GFAP, indicating that most of the Nestin-positive cells were not from reactive astrocytes (Fig. 1M, N). Next, Sox 2, which is essential for maintaining pluripotency in stem cells and highly expressed in NSCs [36, 37], was used to co-label with ASIC1A and Nestin. It showed that nearly 75% ASIC1A- and Nestin-double positive were co-labeled with Sox 2 (Fig. 1O, P). These results indicated that ASIC1A was markedly upregulated in NSCs following SCI.

### ASIC1A activation prevents NSCs from differentiating into oligodendrocytes

To confirm whether ASIC1A expression in NSCs was activated by the extracellular acidic microenvironment within lesion sites following SCI, we first detected the tissue pH alteration in the lesion site (1 cm centered on the epicenter of the compressed lesion) and adjacent segment (1 cm from the cranial edge of lesion site) following SCI by a micro–pH meter. Consistent with previous studies [38, 39], we found a lower tissue pH value in both lesion site and adjacent segment than in sham group and persisted up to 7d following injury (Fig. 2A, B). The lowest pH (6.03 ± 0.06) value was noted in lesion site at 1 dpi (Fig. 2A). Due to the lowest pH value was approaching 6.0, pH 6.0 acidic medium was selected to culture with NSCs to mimic acidic microenvironment. Western blot results showed that the ASIC1A expression level in NSCs peaked at 24 h following exposure to pH 6.0 acidic medium (Fig. 2C, D). Double immunostaining of NESTIN and ASIC1A revealed a significant increase in the percentage of ASIC1A- and NESTIN-positive cells in NSCs treated with pH 6.0 acidic medium for 24 h compared with controls (Fig. 2E, F). To further investigate whether the degree of pH value affect the expression of ASIC1A in NSCs, NSCs were cultured with pH 6.5 acidic medium for 24 h and immunostaining was performed to detect the expression of ASIC1A. As expect, the expression of AISC1A was increased by the treatment of pH 6.5 acidic medium, but this effect was weaker than in pH 6.0 acidic medium-treated NSCs (Fig. 2E, F).

Since NSC differentiation is crucial for myelin sheath regeneration and neurological functional recovery, we examined whether acidic medium affects NSC differentiation. NSCs were digested into single cells and treated with normal culture medium or pH 6.0 acidic medium or pH 6.5 acidic medium for 5 days. Treatment of NSCs with pH 6.0 or pH 6.5 acidic medium resulted in a reduction in the percentage of CNPASE-positive oligodendrocytes and an increase in the proportion of GFAP-positive astrocytes compared with controls (Fig. 2G, H). In contrast, pH 6.0 acidic medium had a stronger effect on promoting the differentiation of NSCs into astrocytes, compared with pH 6.5 acidic medium (Fig. 2G, H). Moreover, to identify whether this acidic medium-induced effect was related to ASIC1A, the addition of the nonspecific ASIC antagonist amiloride and the ASIC1A antagonist psalmotoxin (PcTx) were used to inhibit ASICs and ASIC1A, respectively. As expected, the effect induced by acidic medium on NSC differentiation was abolished by amiloride or PcTx treatment (Fig. 2 G, H). Together, these data indicated that the acidic environment promoted NSC differentiation into astrocytes by upregulating the ASIC1A signaling pathway following SCI.

As extracellular acidification could directly induce apoptosis of cells, we next detected the apoptotic rate of NSCs following exposure to pH 6.0 acidic medium for 24 h by TUNEL staining. It revealed that the treatment of acidic medium significantly increased the apoptotic rate of NSCs compared with controls (Fig. 2I, J). Therefore, there is a possibility that the extracellular acidification might cause distinct apoptotic responses in oligodendrocytes and astrocytes, which resulted into a greater reduction in the proportion of oligodendrocytes during the process of NSC differentiation. To study this possibility, we cultured primary astrocytes and oligodendrocytes and treated these cells with pH 6.0 acidic medium for 24 h respectively. The TUNEL staining results showed that although compared with control groups, the apoptotic rates in oligodendrocytes and astrocytes were marked increased by the treatment of acidic medium, no difference were found between these 2 groups (Fig. 2I, J). It indicated that when facing extracellular acidification, the apoptosis of oligodendrocytes resembled that of astrocytes. These data confirmed that the extracellular acidification induced the reduction in the proportion of oligodendrocytes during the process of NSC differentiation mainly through preventing NSCs differentiation into oligodendrocytes rather than increasing the apoptosis of oligodendrocytes.

### ASIC1A upregulated PTGS2 in acid-treated NSCs and SCI rats

To elucidate the molecular mechanism through which extracellular acidic environment influences the differentiation of NSCs, we conducted RNA-sequence analysis on NSCs treated with pH 6.0 acidic medium. Compared with controls, acidic medium treatment led to significant changes in gene expression, with 42 genes being upregulated and 192 genes being downregulated (Fig. 3A). Among the top ten upregulated and top ten downregulated genes, prostaglandin-endoperoxide synthase 2 (*Ptgs2*) stood out (Fig. 3B), and it is known for its crucial roles in post-trauma inflammation and demyelinating neurological diseases [40–44]. Moreover, KEGG pathway enrichment analysis showed that *ptgs2* were related with the immune-relevant pathways (TNF-signaling pathway and IL-17 signaling pathway, Fig. 3C). By qPCR, we verified that *ptgs2* gene expression was upregulated in NSCs following 24 h of exposure to acidic medium (Fig. 3D). Additionally, acidic medium treatment significantly increased the percentage of PTGS2 and NESTIN double-positive cells suggesting that an acidic environment could upregulate PTGS2 expression in NSCs (Fig. 3E, F). As expected, compared with pH 6.5-acidic medium treated NSCs, pH 6.0-acidic medium revealed a stronger effect on increasing the percentage of PTGS2 and NESTIN double-positive cells (Fig. 3E, F). To identify whether the upregulation of PTGS2 was related to ASIC1A, NSCs were treated with amiloride or PcTx to inhibit the activation of ASIC1A in an acidic environment, and the percentage of PTGS2 and NESTIN double-positive cells was strongly decreased following 24 h of culture compared with acidic medium-treated NSCs (Fig. 3E, F). These data suggested that the upregulation of PTGS2 in NSCs was related to extracellular acidic environment-induced ASIC1A activation. To further investigate whether the acidic environment-induced regulation of NSC differentiation was associated with PTGS2, we added NS398 (PTGS2 inhibitor) to NSCs in an acidic environment. This led to a significant weakening of the acidification-related effect on NSC differentiation, resulting in an increase in the proportion of oligodendrocytes and a reduction in the proportion of astrocytes (Fig. 3G, H). This result indicated that PTGS2 plays a key role in the extracellular acidic environment-induced regulation of NSC differentiation.

To further investigate the relationship between PTGS2 and ASIC1A in SCI rats, we assessed PTGS2 expression in SCI rats at 3 and 7 dpi by immunostaining. The results revealed that PTGS2 expression was significantly increased in the LC and ELC compared to the SNT. Moreover, many of these PTGS2-positive cells were also co-expressed with NESTIN-positive NSCs (Fig. 3I–K), indicating that injury to the spinal cord upregulated PTGS2 expression in NSCs. Next, to confirm whether the upregulation of PTGS2 was related to the activation of ASIC1A, we treated SCI rats with different concentration of amiloride or PcTx by a 3 day continuous intrathecal injection. It revealed that the higher dose of amiloride or PcTx appeared to provide stronger effects in inhibiting the expression of PTGS2 in NSCs at 3 dpi (Fig. 3I, J). No differences were found between the groups treated with amiloride at 100 μM and 200 μM or between the groups treated with PcTx at 10 nM and 20 nM (Fig. 3I, J). Therefore, 100 μM amiloride and 20 nM PcTx were selected for further injection. Similarly, this intervention significantly reduced the proportion of PTGS2-positive NSCs at 7 dpi (Fig. 3I, K). These in vivo results align with the in vitro findings, suggesting that inhibiting the activation of ASIC1A affects the upregulation of PTGS2 in NSCs, strongly indicating that the extracellular acidic environment upregulates PTGS2 expression via ASIC1A.

### The acidic environment regulated NSC differentiation by enhancing transcellular NSC-to-NSC delivery of PGE2

PTGS2 plays a critical role in converting arachidonic acid into prostaglandin H2, which serves as the precursor for various prostaglandins, including prostaglandin E2 (PGE2) [45, 46]. PGE2 is a proinflammatory factor that can be secreted by both nerve and immune cells [47–50]. Additionally, PGE2 has been found to be enriched within extracellular vesicles (EVs) [51, 52], which are membrane-bound particles ranging from 30 to 150 nm and are released by a wide range of cell types [53, 54]. Our recent study has shown that endogenous NSCs can engage in cell-to-cell communication by releasing EVs [33]. Therefore, we hypothesized that in an acidic environment, NSCs release PGE2 within EVs and that these PGE2-enriched EVs regulate the differentiation of NSCs through intercellular communication. To confirm this hypothesis, we first collected EVs from NSCs and confirmed their identity by transmission electron microscopy (TEM), dynamic light scattering and western blot analysis (Fig. 4A–C). Subsequently, the concentration of PGE2 within EVs derived from NSCs treated with either normal medium (nm-NSC-EVs, control groups) or acidic medium (am-NSC-EVs, pH 6.0 groups) was determined using ELISA. Compared with untreated NSCs (controls), exposure to acidic medium at pH 6.0 significantly increased the concentration of PGE2 within EVs (Fig. 4D).

Since Pge2 facilitates signals by binding to specific cell surface prostaglandin E receptors (Ep1-Ep4) [55–57] and EP2 has been reported to be widely expressed in the central nervous system (CNS) [46, 58, 59], we then detected whether extracellular acidification could activate EP2 in NSCs. Following 24 h of treatment with acidic medium, immunostaining revealed that EP2 was colocalized with Nestin-positive NSCs, and the percentage of NESTIN- and EP2-double-positive cells markedly increased compared with controls (Fig. 4 E, F). Next, to confirm whether the upregulation of PGE2 and its related receptor EP2 were induced by activation of ASIC1A, we added PcTx to NSCs in an acidic environment. We then detected the concentration of PGE2 within NSC-EVs by ELISA and calculated the proportion of NESTIN- and EP2- double-positive cells by immunostaining. As expected, treatment with PcTx strongly reduced the concentration of PGE2 within EVs (Fig. 4D) and the percentage of double-positive cells compared to those exposed to acidic treatment at pH 6.0 (Fig. 4E, F). Moreover, in vivo, PcTx treatment reduced the expression of EP2 in NESTIN-positive NSCs in the LC and ELC at 3 and 7 dpi (Fig. 4G–I). These data indicated that acidic-dependent ASIC1A enhances the expression of PGE2/EP2.

To further investigate whether this ASIC1A-induced upregulation of PGE2 in NSC-EVs was associated with the regulation of NSC differentiation, we first treated NSCs with AH-6809 (a prostaglandin E2 receptor antagonist) in an acidic environment. This led to an increase in the percentage of CNPASE-positive cells compared with NSCs cultured with pH 6.0 acidic medium (Fig. 4J, K). To identify whether these acidic environment-induced alterations in NSC differentiation were related to NSC-EVs, we labeled NSC-EVs with Pkh-26 and treated NSCs and SCI rats with these Pkh-26-tracked NSC-EVs. Following a 24 h coculture with NSCs, it was discovered that labeled EVs (red) were present in the cytoplasm near the nucleus of NSCs (Fig. 4L). In vivo, following the intrathecal injection of Pkh-26-tracked NSC-EVs into SCI rats, immunostaining showed that these Pkh-26-tracked NSC-EVs were contained within the cytoplasm of these Nestin-positive cells (Fig. 4M).

Next, we collected EVs from normal medium-treated NSCs (nm-NSC-EVs) and acidic medium-treated NSCs (am-NSC-EVs), respectively, and treated NSCs with PGE2, nm-NSC-EVs and am-NSC-EVs for 5 days, and assessed the proportion of CNPASE-positive oligodendrocytes by immunostaining. Compared with the controls, the addition of nm-NSC-EVs significantly increased the proportion of CNPASE-positive cells (Fig. 4N, O). In contrast, the addition of PGE2 or am-NSC-EVs reduced the proportion of CNPASE-positive cells (Fig. 4N, O). Subsequently, we added PGE2 to NSCs in the presence of nm-NSC-EVs and AH-6809 to NSCs in the presence of am-NSC-EVs and showed that the addition of Pge2 abolished the nm-NSC-EV-induced effects on the differentiation of NSCs, resulting in a reduction in the proportion of CNPASE-positive cells (Fig. 4N, O). As expected, the addition of AH-6809 blocked the am-NSC-EV-induced effects and led to an increase in the percentage of CNPASE-positive cells (Fig. 4N, O). All these data indicate that the ASIC1A-induced upregulation of PGE2 within NSC-EVs plays a key role in preventing NSC differentiation toward oligodendrocytes.

### Ptgs2 or Asic1a deficiency in NSCs countered acidic environment-induced effects on NSC differentiation by reducing the transcellular delivery of PGE2

To further confirm whether the am-NSC-EV-induced effect on mediating NSC differentiation was associated with PTGS2, siRNA-Ptgs2 (si-Ptgs2) was used to knock down Ptgs2 expression in NSCs (Fig. 5A). Compared with am-NSC-EVs, the concentration of PGE2 within the EVs derived from si-Ptgs2-transfected NSCs exposed to acidic medium (am-NSC^si-Ptgs2^-EVs) was markedly reduced (Fig. 5B). As expected, am-NSC^si-Ptgs2^-EVs lost their ability to regulate NSC differentiation, leading to an increase in the proportion of CNPASE-positive cells compared with NSCs treated with am-NSC-CM (Fig. 5C, D).

Since the upregulation of PTGS2/PGE2 was induced by the activation of ASIC1A, we used Asic1a-siRNA to knock down Asic1a expression in NSCs (Fig. 5E). In comparison with am-NSC-EVs, treatment with acidic medium did not enhance the concentration of PGE2 within EVs derived from si-Asic1a-transfected NSCs (Fig. 5F). Similarly, these EVs derived from am-treated NSC^si-Asic1a^ (am-NSC^si-Asic1a^-EVs) resulted in an increase in the percentage of CNPASE-positive cells compared with am-NSC-EV-treated NSCs (Fig. 5G, H). All these findings indicated that the Asic1a-related delivery of PGE2 played a crucial role in preventing NSCs from differentiating into oligodendrocytes.

### PcTx promoted myelin sheath regeneration and axonal remyelination in lesion sites

To evaluate the effects of PcTx on SCI rats, we first detected the regeneration of the myelin sheath at week 2 post-injury. Immunostaining showed that in SCI rats, a cavity formed in the center of the lesion site, surrounded by a GFAP-positive astrocytic boundary. Few CNPASE-positive cells were found within this astrocytic boundary and cavity. Rats injected with PcTx exhibited a significant increase in the percentage of CNPASE-positive areas (Fig. 6A, B), indicating enhanced myelin sheath regeneration.

As the corticospinal tract is of the utmost importance for the recovery of impaired motor function following SCI [60, 61], we detected axonal remyelination in the lateral corticospinal tract at week 4 post-injury by immunostaining with a mature oligodendrocyte marker, MBP. In SCI rats, regenerated TUJ1-positive neuronal fibers were observed around the cavity. However, compared with Sham rats, in which TUJ1-positive fibers were ensheathed with MBP-positive myelin sheets, the intensity of MBP-positive myelin within axons was significantly reduced in SCI rats (Fig. 6C, D), indicating demyelination in the axons. In contrast, injection of PcTx markedly increased the intensity of MBP-positive myelin sheets within the TUJ-positive areas (Fig. 6C, D), indicating that the injection of PcTx promoted axonal remyelination in lesion sites following SCI. The neurological results were consistent with the histological findings. The rats that received PcTx injection exhibited a better outcome in both BBB scores and angle of inclined plane test (Fig. 6E, F), indicating improved neurological functional recovery following SCI. All these data suggested that the PcTx treatment promoted myelin sheath regeneration and axonal remyelination, resulting in an improvement of the neurological functional recovery following SCI.

### Asic1a-KO mice fail to upregulate PTGS2 expression, resulting in an improvement in axonal remyelination following SCI

To further study the role of ASIC1A in SCI and its relationship with PTGS2, Asic1a knockout (Asic1a-KO) mice were used (Fig. 7A). Following SCI in Asic1a-KO mice, PTGS2 expression in NSCs remained at a low level compared to that in wild-type (WT) mice (Fig. 7B, C). Next, NSCs were obtained from WT and Asic1a-KO mice and treated with acidic medium at pH 6.0, and their EVs were collected. Immunostaining showed that NSCs obtained from Asic1a-KO mice had a complete loss of Asic1a expression (Fig. 7D) and a lower percentage of PTGS2-positive cells than NSCs collected from WT mice (Fig. 7E, F). The ELISA results showed that the concentration of PGE2 was markedly lower in the EVs derived from Asic1a-KO NSCs (Fig. 7G). All these findings indicated that ASIC1A was needed for the upregulation of PTGS2 in response to the acidic environment.

To investigate the role of ASIC1A in NSC differentiation and axonal remyelination, NSCs obtained from WT mice and Asic1a-KO mice received acidic medium for 5 days. Immunostaining showed that Asic1a-KO NSCs had a higher percentage of CNPASE-positive NSCs than WT NSCs (Fig. 7H, I). Moreover, this Asic1a-KO-induced increase was abolished by the addition of PGE2, resulting in a decrease in the percentage of CNPASE-positive cells (Fig. 7H, I). These findings strongly indicated that PTGS2 played a key role in ASIC1A-induced mediation of NSC differentiation. At the edge of the lesion core, the total TUJ1 and MBP double-positive areas were significantly higher in Asic1a-KO mice than in WT mice at 4 weeks post-injury (Fig. 7J, K). Consistent with the histology results, Asic1a-KO mice exhibited better outcomes in both BBB scores and the inclined plane test (Fig. 7J, K). This highlighted that Asic1a deficiency enhanced axonal remyelination, leading to neurological recovery.

## Discussion

**Fig. 8:**
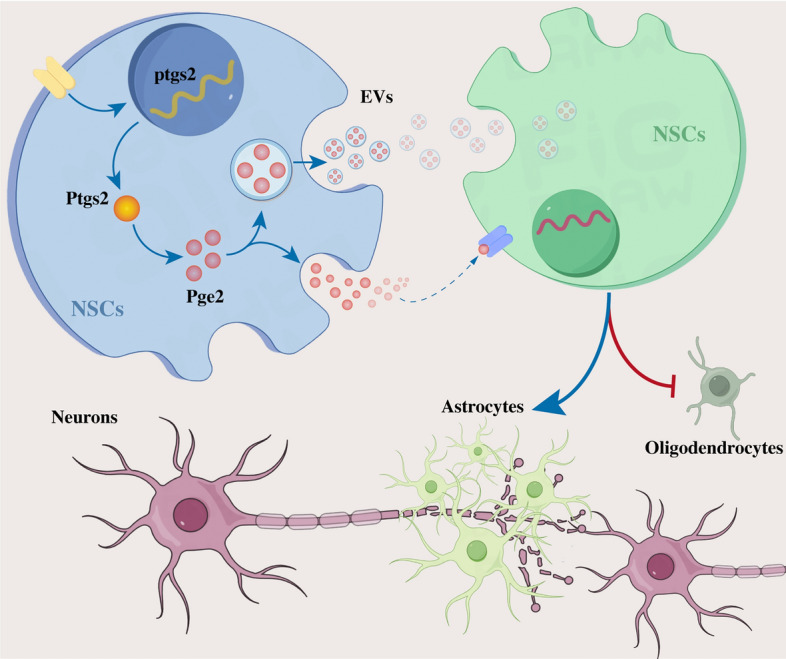
Acidic environment Induces transcellular NSC–NSC delivery of PGE2 regulating NSC Differentiation through the ASIC1A/PTGS2 axis

In the present study, we elucidate an acidic environment-induced ASIC1A/PTGS2/PGE2 signaling axis that prevents NSC differentiation into oligodendrocytes by the trans-cellular delivery of PGE2, thereby contributing to the failure of myelin sheath regeneration after SCI. Repressing the activation of ASIC1A inhibits PGE2-induced regulation of NSC differentiation enhances regeneration of the myelin sheath and improves axonal remyelination. Our findings indicated the efficacy of mitigating the impact of acidic environments on NSCs in facilitating neurological recovery after SCI (Fig. 8).

Tissue acidosis within lesion sites is a common phenomenon that occurs following CNS damage. Emerging evidence has shown that a decrease in pH may directly regulate the inflammatory process, oxidative stress [62], and bioactivities of nerve cells by activating ASICs. Our findings indicated that the acidosis-related ASIC1A was widespread in endogenous NSCs, indicating that NSCs were influenced by the acidic environment in lesion sites following SCI. Acidic treatment of NSCs upregulated ASIC1A expression, leading to a decline in the percentage of oligodendrocytes. Furthermore, repression of ASIC1A countered these acidic medium-induced effects. Additionally, the injection of the ASIC1A antagonist PcTx following SCI promoted regeneration of the myelin sheath and axonal remyelination, resulting in an improvement in neurological recovery. Moreover, compared with the SCI WT mice, the Asic1a-KO mice exhibited an improvement in axonal myelination following SCI. All these data highlight that the acidic environment-induced influence on NSCs might contribute to the failure of myelin sheath regeneration through activating ASIC1A.

As repressing the activation of ASIC1A resulted in a better histological and neurological improvement, ASIC1A might serve as a promising therapeutic target for SCI. These results were consistent with previous studies [20, 23]. It was reported that the upregulation of ASIC1A was associated with the delayed neuronal death following SCI and suppressing ASIC1a attenuated the death of spinal cells leading to the improvement of neurological recovery [20]. Similarly, the treatment of PcTx was also reported to achieve an improvement of neurological recovery following SCI by reducing the extent of inflammation-related secondary tissue loss [23]. Moreover, in other pathological contexts, the inhibition of ASIC1A also achieved beneficial effects through mediating inflammatory process. For instance, ASIC1A blockers could significantly enhance clinical scores in experimental autoimmune encephalomyelitis (EAE) mice by offering neuroprotection [14]. According to a study on rheumatoid arthritis (RA), extracellular acidification induced the upregulation of ASIC1A leading to the exacerbation of synovial inflammation, while this inflammation-related arthritic severity could be alleviated by repressing the expression of ASIC1A [15]. Taken together, all these findings indicated that ASIC1A was a promising therapeutic target for alleviating acidosis-induced detrimental impacts.

PTGS2, an enzyme responsible for converting arachidonic acid into prostaglandin H2, is recognized as the precursor of numerous other prostaglandins and their derivatives, including PGD, PGE, PGF, PGI, and thromboxanes [45, 49, 50]. It is well known as an inflammatory mediator that directly inhibits the maturation of oligodendrocyte progenitor cells and is associated with the aggravation of white matter injury [50]. In the present study, we observed that the PTGS2 expression levels in NSCs was significantly elevated in acidic environment. Moreover, both in vitro acidic medium-induced and in vivo spinal cord injury (SCI)-induced upregulation of PTGS2 were substantially attenuated upon the administration of ASIC1A-related antagonists. More importantly, compared with WT NSCs, Asic1a-KO NSCs failed to upregulate PTGS2 expression after acidic treatment. Similarly, Asic1a-KO mice exhibited lower PTGS2 expression levels in lesion sites following SCI than WT mice. These data suggested that the upregulation of PTGS2 expression induced by an acidic environment is closely associated with ASIC1A activation. Furthermore, multiple lines of evidence support the crucial role of PTGS2 in the regulation of NSC differentiation. First, PGE2 mimicked the effects of acidic treatment by preventing NSCs from differentiating into oligodendrocytes. Second, treatment with PTGS2 selective inhibitors or prostaglandin E2 receptor antagonists effectively abolished the acidic treatment-induced modulation of NSC differentiation. Finally, the addition of PGE2 to Asic1a-KO NSCs restored the acidic environment-induced effects on NSC differentiation. These findings demonstrated that extracellular acidification mediates NSC differentiation through the ASIC1A/PTGS2/PGE2 axis.

EVs are membrane-delimited particles encapsulating proteins, microRNAs, and lipids that play a key role in regulating multiple pathological processes through intercellular communication [63–65]. Following neurotrauma, EVs contribute to the regulation of multiple pathologies by carrying parent cell-specific cargoes that modify the function of recipient cells [66–68]. Moreover, in response to external microenvironmental stimuli, the bioactivity and cargoes of EVs can vary [32, 69, 70]. In our recent studies, we pointed out that inflammatory stimulation of astrocytes could enhance the bioeffects of NSC-derived EVs on axonal regeneration and remyelination, whereas EVs originating from inflammation-stimulated neurons hinder axonal remyelination following SCI [33]. In the present study, we revealed that compared with NSC-EVs, whose bioeffects promoted NSC differentiation into oligodendrocytes, EVs from acidic medium-treated NSCs lost this bioactivity, switching to inhibiting NSC differentiation into oligodendrocytes. Subsequent data demonstrated the pivotal role of PGE2 in this transformation of biological functionality. First, the concentration of PGE2 within EVs was upregulated by the treatment of acidic medium, and this upregulation could be reversed by the addition of an ASIC1A antagonist or the knockdown of Asic1a. Second, the bioeffects of NSC-EVs on NSC differentiation were associated with the concentration of PGE2 within EVs. Specifically, NSCs exposed to acidic medium, which had a high PGE2 concentration within their released EVs, exerted a stronger effect on repressing NSC differentiation into oligodendrocytes than normal medium-treated NSCs. Moreover, elevating the PGE2 concentration within EVs derived from normal medium-treated NSCs transformed the bioeffects of these EVs from promotion to inhibition of NSC differentiation into oligodendrocytes.

## Limitation

In present study, to create a severe crush SCI model, only female animals were utilized due to the ease of urine evacuation that could avoid urinary infections and hematuria [71]. It remains unclear whether the difference of gender affects the expression of ASIC1A and ASIC1A-related PTGS2. In addition, the impact of this gender differences on the effects of ASIC1A blockers in enhancing neurological recovery is still unclear.

## Conclusion

The acidic environment related activation of ASIC1A modulates NSC differentiation by enhancing the trans-cellular delivery of PGE2. The inhibition of ASIC1A presents a promising therapeutic strategy for enhancing myelin sheath regeneration and promoting axonal remyelination following SCI.

## Data Availability

The datasets used and/or analyzed during the current study are available from the corresponding author upon reasonable request.

## Abbreviations

SCI: Spinal cord injury
NSCs: Neural stem cells
CNS: Central nervous system
ASICs: Acid-sensing ion channels
PTGS2: Prostaglandin endoperoxide synthase 2
PGE2: Prostaglandin E2
EVs: Extracellular vesicles
GFAP: Glial fibrillary acidic protein
TUJ-1: Neuron-specific class III beta-tubulin
CNPASE: 2′3′ cyclic nucleotide 3′ phosphodiesterase
MBP: Myelin basic protein
